# Supplementary material for: Differential Sensitivity to MEK Inhibitors Highlights Distinct Entosis Mechanisms in BxPC3 and MCF7 Cells
Source: Cells. 2025 Sep 25;14(19):1500. doi: 10.3390/cells14191500 (PMC12524069; doi:10.3390/cells14191500)
Supplement: Supplementary file 1 [file cells-14-01500-s001.zip › cells-3863843-supplementary.pdf]

Supplementary Fig S1:

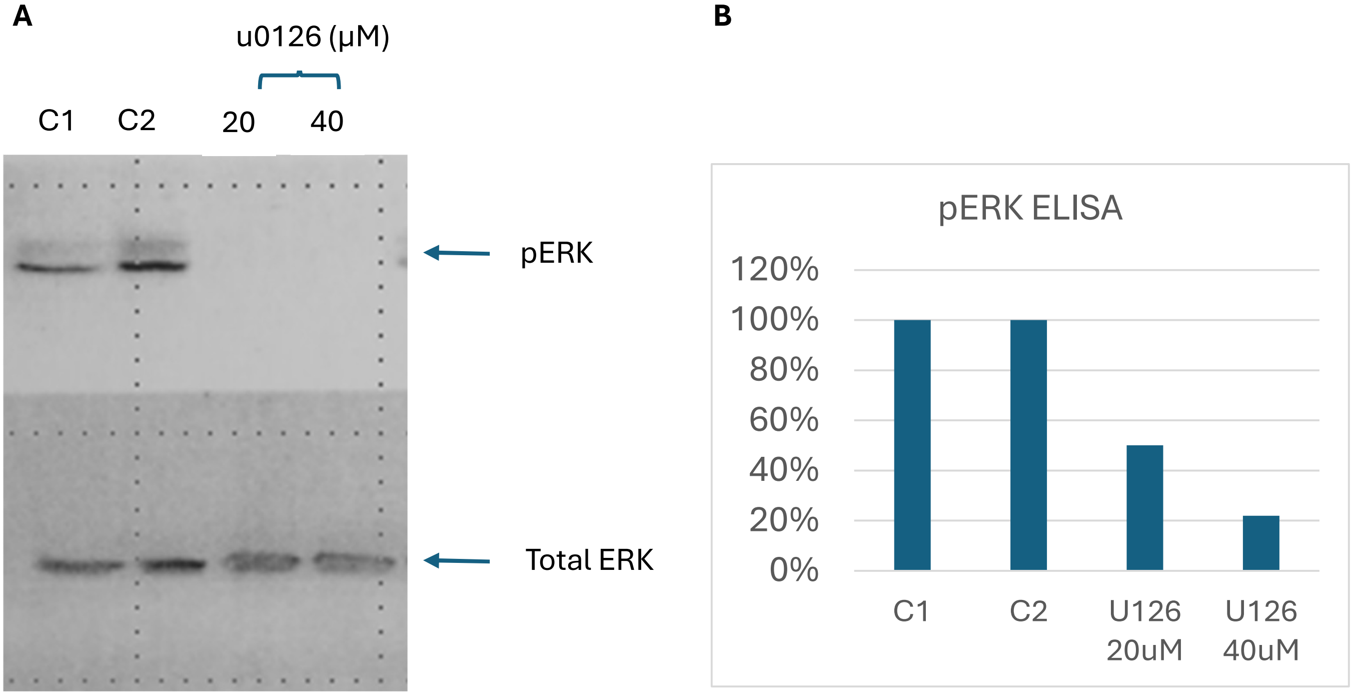

A: Western blot for pERK (upper panel) and total ERK (lower panel) in BxPC3 cells: C1 and C2 are two independent controls, lanes 3 and 4 – cells were treated with U126 20 μM and 40 μM for 24h.

B: pERK ELISA assay of the same samples as in lines 1-4 from WB.

Supplementary Fig S2:

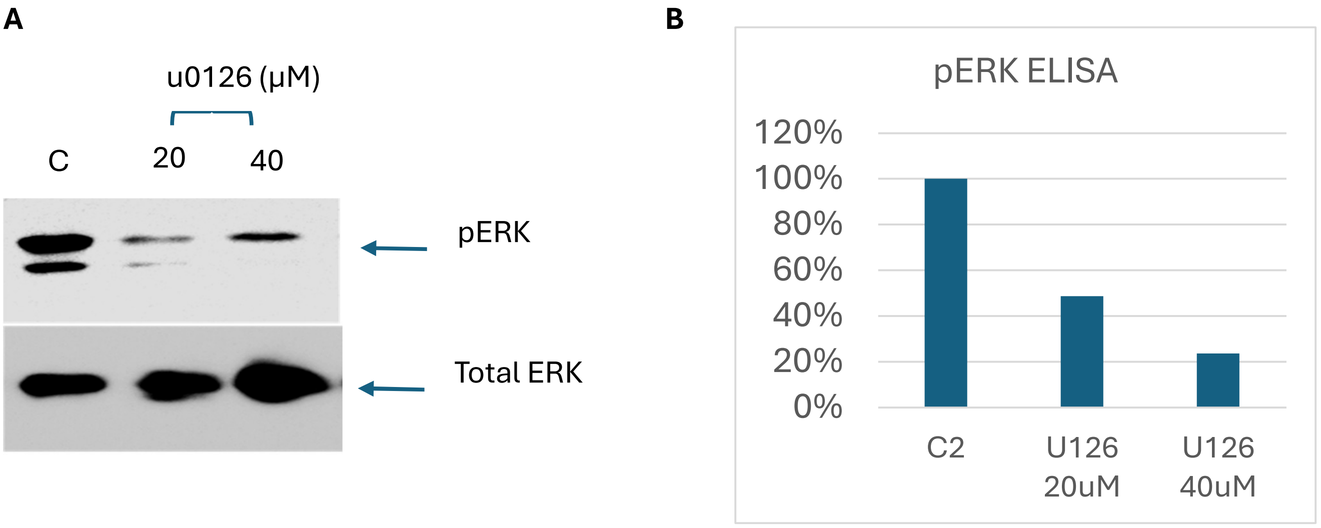

A: Western blot for pERK (upper panel) and total ERK (lower panel) in MCF7 cells: C, control, lanes 2 and 3 – cells were treated with U126 10 μM and 40 μM for 24h.

B: pERK ELISA assay of the same samples as in lines 1-4 from WB.
